# Supplementary material for: Association between NTRK2 Polymorphisms, Hippocampal Volumes and Treatment Resistance in Major Depressive Disorder
Source: Genes (Basel). 2023 Nov 3;14(11):2037. doi: 10.3390/genes14112037 (PMC10671548; doi:10.3390/genes14112037)
Supplement: Supplementary file 1 [file genes-14-02037-s001.zip › genes-2667863-supplementary.pdf]

Supplementary Material

Supplementary Table S1: Polymorphisms distribution in the study sample.

Variant name	Gene (Chr.)	Alleles	AA (n. subj)	AB (n. subj)	BB (n. subj)	MapInfo
rs1187350	NTRK2 (9)	[A/G]	29	65	27	87295237
rs1619120	NTRK2 (9)	[A/G]	23	57	41	87302196
rs1187343	NTRK2 (9)	[A/G]	37	64	19	87308783
rs1187337	NTRK2 (9)	[T/C]	27	64	30	87316037
rs11140745	NTRK2 (9)	[T/C]	12	55	54	87355358
rs1573219	NTRK2 (9)	[A/G]	10	56	54	87387622
rs1443440	NTRK2 (9)	[T/C]	69	43	7	87406168
rs1899640	NTRK2 (9)	[A/G]	53	56	12	87409025
rs1187287	NTRK2 (9)	[A/G]	3	42	76	87414794
rs1187286	NTRK2 (9)	[T/G]	64	49	8	87415028
rs716893	NTRK2 (9)	[A/G]	14	55	52	87419117
rs3739804	NTRK2 (9)	[A/G]	97	23	1	87421631
rs10512154	NTRK2 (9)	[A/G]	6	50	65	87441475
rs2083828	NTRK2 (9)	[A/C]	20	65	36	87447045
rs7855888	NTRK2 (9)	[T/C]	68	47	6	87452058
rs1838158	NTRK2 (9)	[A/G]	96	24	1	87473009
rs10116287	NTRK2 (9)	[T/G]	79	35	7	87477274
rs7048015	NTRK2 (9)	[A/C]	92	27	2	87478135
rs10780691	NTRK2 (9)	[T/C]	31	66	24	87491253
rs10868238	NTRK2 (9)	[T/C]	18	65	38	87530935
rs12340212	NTRK2 (9)	[A/G]	0	26	95	87551964
rs6559838	NTRK2 (9)	[T/C]	2	31	88	87553563
rs2808707	NTRK2 (9)	[T/G]	19	62	40	87558294
rs6559840	NTRK2 (9)	[T/C]	4	45	72	87575500
rs3860945	NTRK2 (9)	[A/G]	87	33	1	87585624
rs4877894	NTRK2 (9)	[A/G]	40	64	17	87590382
rs10868241	NTRK2 (9)	[A/G]	4	54	63	87593028
rs4361832	NTRK2 (9)	[A/G]	2	44	75	87595734
rs1948308	NTRK2 (9)	[A/G]	43	57	21	87616257
rs923559	NTRK2 (9)	[A/G]	4	50	67	87616532
rs1387924	NTRK2 (9)	[T/G]	1	40	80	87632993
rs10835189	BDNF (11)	[T/G]	30	70	21	27541995
rs7127239	BDNF (11)	[A/C]	0	29	92	27547379
exm-rs988712	BDNF (11)	[T/G]	12	49	60	27563382
exm-rs7481311	BDNF (11)	[T/C]	0	30	91	27583129
rs10835201	BDNF (11)	[A/G]	91	30	0	27618265
rs10734394	BDNF (11)	[A/G]	60	49	12	27628412
rs1387144	BDNF (11)	[A/C]	48	57	16	27635319

rs6416056	BDNF (11)	[A/G]	54	50	17	27646745
rs4074134	BDNF (11)	[T/C]	9	47	65	27647285
exm-rs925946	BDNF (11)	[T/G]	3	34	84	27667202
rs1519480	BDNF (11)	[T/C]	79	39	3	27675712
rs6265	BDNF (11)	[T/C]	7	40	74	27679916
rs10835210	BDNF (11)	[A/C]	27	57	34	27695910
rs10835211	BDNF (11)	[A/G]	2	30	89	27701365
exm-rs10767664	BDNF (11)	[A/T]	62	44	12	27725986
rs2030323	BDNF (11)	[A/C]	13	45	63	27728539
rs7934165	BDNF (11)	[A/G]	33	60	28	27731983

Supplementary Table S2: effect of polymorphisms on Hippocampal volumes and treatment resistance. Statistical significance (p) reported.

Variant name	Genetic model	Hippocampus, right (p)	Hippocampus, left (p)	Treatment resistance (p)
rs1187350	AA vs AB + BB	0.12881647	0.13746592	0.81797011
	AA + AB vs BB	0.61453020	0.50403903	0.87007879
	AA + BB vs AB	0.38409898	0.48089286	0.95212864
rs1619120	AA vs AB + BB	0.90512449	0.53026059	0.43954745
	AA + AB vs BB	0.06137145	0.02992682	0.55504501
	AA + BB vs AB	0.09409391	0.12104164	0.96215809
rs1187343	AA vs AB + BB	0.05056225	0.06276421	0.59114335
	AA + AB vs BB	0.08362525	0.04069298	0.42065316
	AA + BB vs AB	0.59789403	0.82961192	0.92286221
rs1187337	AA vs AB + BB	0.38338555	0.18610124	0.83717457
	AA + AB vs BB	0.05689802	0.04652495	0.63953707
	AA + BB vs AB	0.36604759	0.54669268	0.56537834
rs11140745	AA vs AB + BB	0.82586438	0.76690087	0.21701167
	AA + AB vs BB	0.40765706	0.88056239	0.92358819
	AA + BB vs AB	0.48849884	0.97601280	0.51851537
rs1573219	AA vs AB + BB	0.46873208	0.34742606	0.47448297
	AA + AB vs BB	0.22875811	0.67148275	0.93795238
	AA + BB vs AB	0.42892147	0.91814876	0.74839792
rs1443440	AA vs AB + BB	0.81231725	0.93743066	0.18120329
	AA + AB vs BB	0.34902998	0.23668678	0.19352706
	AA + BB vs AB	0.82100118	0.60734721	0.46472164
rs1899640	AA vs AB + BB	0.21975924	0.70057145	0.95497601
	AA + AB vs BB	0.72336955	0.95558686	0.55817769
	AA + BB vs AB	0.31586389	0.72761492	0.68362160
rs1187287	AA vs AB + BB	0.52108265	0.49261727	0.56021958
	AA + AB vs BB	0.56289134	0.66685098	0.42590558
	AA + BB vs AB	0.42340588	0.50638406	0.53577933
rs1187286	AA vs AB + BB	0.31061448	0.60167944	0.66431553
	AA + AB vs BB	0.55732905	0.46304479	0.98628843
	AA + BB vs AB	0.46367785	0.87621602	0.66491313
rs716893	AA vs AB + BB	0.75973033	0.50061795	0.08103649
	AA + AB vs BB	0.77193339	0.90528601	0.57040382
	AA + BB vs AB	0.62390692	0.57362650	0.08775880
rs3739804	AA vs AB + BB	0.87344608	0.76299037	0.57208665
	AA + AB vs BB	0.56681855	0.75354214	-
	AA + BB vs AB	0.90016046	0.81366619	0.74376451
rs10512154	AA vs AB + BB	0.36047348	0.05110025	0.07887819
	AA + AB vs BB	0.53826500	0.40386930	0.72550695
	AA + BB vs AB	0.28966741	0.07702177	0.26098935
rs2083828	AA vs AB + BB	0.81044504	0.77174660	0.97975878

	AA + AB vs BB	0.51604190	0.48180596	0.45837551
	AA + BB vs AB	0.68290057	0.67463674	0.48423824
rs7855888	AA vs AB + BB	0.02033028	0.00923203	0.84410085
	AA + AB vs BB	0.44823561	0.61751472	0.94127165
	AA + BB vs AB	0.00675813	0.00399182	0.86736039
rs1838158	AA vs AB + BB	0.81759058	0.64582867	0.04324420
	AA + AB vs BB	0.48817413	0.17094599	-
	AA + BB vs AB	0.93903970	0.87609723	0.02169181
rs10116287	AA vs AB + BB	0.02430848	0.03127852	0.14944726
	AA + AB vs BB	0.21583179	0.34308771	0.65434317
	AA + BB vs AB	0.09145789	0.08228071	0.08288701
rs7048015	AA vs AB + BB	0.93944470	0.34608860	0.60548157
	AA + AB vs BB	0.80628848	0.63316810	-
	AA + BB vs AB	0.87737326	0.26625216	0.93870674
rs10780691	AA vs AB + BB	0.16842735	0.13726942	0.84006786
	AA + AB vs BB	0.20433280	0.24990255	0.69297711
	AA + BB vs AB	0.84784974	0.70358912	0.89017694
rs10868238	AA vs AB + BB	0.61620071	0.94488637	0.29548969
	AA + AB vs BB	0.17104885	0.22804506	0.41370905
	AA + BB vs AB	0.35757763	0.28282214	0.13436442
rs12340212	AA vs AB + BB	-	-	-
	AA + AB vs BB	0.33144622	0.20611303	0.01259715
	AA + BB vs AB	-	-	-
rs6559838	AA vs AB + BB	0.38872541	0.08194939	-
	AA + AB vs BB	0.10909464	0.29673016	0.12290434
	AA + BB vs AB	0.16790988	0.57465986	0.27645329
rs2808707	AA vs AB + BB	0.18307919	0.30822029	0.39250288
	AA + AB vs BB	0.28912152	0.73701278	0.71739129
	AA + BB vs AB	0.95474247	0.68288521	0.78998991
rs6559840	AA vs AB + BB	0.16615199	0.37674664	0.98828254
	AA + AB vs BB	0.91276063	0.84896404	0.63549670
	AA + BB vs AB	0.67827500	0.59873573	0.63526987
rs3860945	AA vs AB + BB	0.90400950	0.98155510	0.59682443
	AA + AB vs BB	0.46186668	0.84954047	-
	AA + BB vs AB	0.97681063	0.95026456	0.76571676
rs4877894	AA vs AB + BB	0.00429649	0.04341798	0.10486081
	AA + AB vs BB	0.20431480	0.43566178	0.61984364
	AA + BB vs AB	0.00025793	0.01310722	0.05959438
rs10868241	AA vs AB + BB	0.82082463	0.70970832	0.96184390
	AA + AB vs BB	0.11505766	0.30581381	0.09339024
	AA + BB vs AB	0.13300534	0.37065268	0.09471939
rs4361832	AA vs AB + BB	0.54986351	0.90109945	-
	AA + AB vs BB	0.03848347	0.21620810	0.31263665
	AA + BB vs AB	0.05323393	0.19793818	0.31753188
rs1948308	AA vs AB + BB	0.00724631	0.05517540	0.01097101
	AA + AB vs BB	0.09557664	0.11551445	0.05212992
	AA + BB vs AB	**0.00008164**	0.00202752	**0.00007461**
rs923559	AA vs AB + BB	0.61500825	0.84861136	0.28726704
	AA + AB vs BB	0.04735609	0.12547950	0.01329093
	AA + BB vs AB	0.02787157	0.10420068	0.00385164
rs1387924	AA vs AB + BB	0.51352646	0.81410576	-
	AA + AB vs BB	0.02176759	0.12020412	0.10507655
	AA + BB vs AB	0.02937639	0.10749887	0.06911655
rs10835189	AA vs AB + BB	0.07797557	0.15100154	0.70057082
	AA + AB vs BB	0.85825516	0.79540367	0.78304676
	AA + BB vs AB	0.16494460	0.29583741	0.90026863
rs7127239	AA vs AB + BB	-	-	-
	AA + AB vs BB	0.04963697	0.10503677	0.36278078
	AA + BB vs AB	-	-	-
exm-rs988712	AA vs AB + BB	0.36167915	0.36823156	0.58996994
	AA + AB vs BB	0.95845898	0.79780070	0.31797526
	AA + BB vs AB	0.61811543	0.42089664	0.48940691
exm-rs7481311	AA vs AB + BB	-	-	-
	AA + AB vs BB	0.11942532	0.23322491	0.47937436
	AA + BB vs AB	-	-	-
rs10835201	AA vs AB + BB	0.11942532	0.23322491	0.47937436
	AA + AB vs BB	-	-	-
	AA + BB vs AB	-	-	-

rs10734394	AA vs AB + BB	0.95845898	0.79780070	0.31797526
	AA + AB vs BB	0.60142087	0.61984827	0.58759876
	AA + BB vs AB	0.79163455	0.57358693	0.49068007
rs1387144	AA vs AB + BB	0.12046321	0.05960440	0.23733065
	AA + AB vs BB	0.46911116	0.76096103	0.62716773
	AA + BB vs AB	0.04144863	0.03838271	0.13459470
rs6416056	AA vs AB + BB	0.17035485	0.17960163	0.57385751
	AA + AB vs BB	0.85152197	0.83694733	0.83650955
	AA + BB vs AB	0.12785784	0.13191716	0.47395732
rs4074134	AA vs AB + BB	0.90594925	0.95696991	0.30997234
	AA + AB vs BB	0.47191756	0.37323237	0.61934701
	AA + BB vs AB	0.42354483	0.34689753	0.97418477
exm-rs925946	AA vs AB + BB	0.94802551	0.37869057	0.69513768
	AA + AB vs BB	0.82458435	0.69989164	0.18807966
	AA + BB vs AB	0.83589378	0.92231951	0.13725887
rs1519480	AA vs AB + BB	0.57285281	0.47676941	0.02595799
	AA + AB vs BB	0.94802551	0.37869057	0.69513768
	AA + BB vs AB	0.54723065	0.30622983	0.01589717
rs6265	AA vs AB + BB	0.84235532	0.84386217	0.22740261
	AA + AB vs BB	0.81089678	0.45919276	0.49774485
	AA + BB vs AB	0.72890276	0.38690601	0.91024038
rs10835210	AA vs AB + BB	0.18641017	0.20845558	0.47168931
	AA + AB vs BB	0.34614058	0.20825930	0.08974604
	AA + BB vs AB	0.79926828	0.93465778	0.35596612
rs10835211	AA vs AB + BB	0.98332878	0.61103824	-
	AA + AB vs BB	0.84153351	0.75952447	0.47686896
	AA + BB vs AB	0.84239760	0.64440750	0.49972208
exm-rs10767664	AA vs AB + BB	0.66253113	0.54159227	0.86381979
	AA + AB vs BB	0.64227193	0.79976920	0.60495442
	AA + BB vs AB	0.87246580	0.63674106	0.88463239
rs2030323	AA vs AB + BB	0.63738662	0.72381612	0.76598851
	AA + AB vs BB	0.67425113	0.51526175	0.92798032
	AA + BB vs AB	0.89583978	0.65659975	0.92210801
rs7934165	AA vs AB + BB	0.22974800	0.30059855	0.25988626
	AA + AB vs BB	0.85988862	0.50969048	0.41486009
	AA + BB vs AB	0.35958768	0.72004069	0.75594346

Supplementary Table S3: details on prescribed pharmacotherapies.

**Whole sample** (n=121)	
SSRIs	74
SNRIs	35
TCAs	12
**Add-on treatments** (n=31)	
Amisulpride (25-50 mg/die)	11
Mirtazapine (15-30 mg/die)	16
Low dosage antipsychotic	3
Mood stabilizer	1
